# Effects of Dynamic Crosslinking on Crystallization, Structure and Mechanical Property of Ethylene-Octene Elastomer/EPDM Blends

**DOI:** 10.3390/polym14010139

**Published:** 2021-12-30

**Authors:** Yuan-Xia Wang, Chen-Chen Wang, Ying Shi, Li-Zhi Liu, Nan Bai, Li-Fu Song

**Affiliations:** 1 Advanced Manufacturing Institute of Polymer Industry, Shenyang University of Chemical Technology, Shenyang 110142, China; wangyuanxia@aliyun.com (Y.-X.W.); wangchenchne@126.com (C.-C.W.); violetlj@yahoo.com (L.-Z.L.); nanbaiasir@126.com (N.B.); Songli_fu@163.com (L.-F.S.); 2 College of Materials Science and Engineering, Shenyang University of Chemical Technology, Shenyang 110142, China

**Keywords:** dynamic crosslinking, ethylene-octene elastomer, compatibility, crystallization behavior, mechanical properties

## Abstract

The dynamic crosslinking method has been widely used to prepare rubber/plastic blends with thermoplastic properties, and the rubber phase is crosslinked in these blends. Both polyolefin elastomer (POE) and ethylene-propylene-diene monomer rubber (EPDM) can be crosslinked, which is different from usual dynamic crosslinking components. In this paper, dynamic crosslinked POE/EPDM blends were prepared. For POE/EPDM blends without dynamic crosslinking, EPDM can play a nucleation role, leading to POE crystallizing at a higher temperature. After dynamic crosslinking, the crosslinking points hinder the mobility of POE chains, resulting in smaller crystals, but having too many crosslinking points suppresses POE crystallization. Synchrotron radiation studies show that phase separation occurs and phase regions form in non-crosslinked blends. After crosslinking, crosslinking points connecting EPDM and part of POE chains, enabling more POE to enter the EPDM phase and thus weakening phase separation, indicates that dynamic crosslinking improves the compatibility of POE/EPDM, also evidenced by a lower β conversion temperature and higher α conversion temperature than neat POE from dynamic mechanical analysis. Moreover, crosslinking networks hinder the crystal fragmentation during stretching and provide higher strength, resulting in 8.3% higher tensile strength of a 10 wt% EPDM blend than neat POE and almost the same elongation at break. Though excessive crosslinking points offer higher strength, they weaken the elongation at break.

## 1. Introduction

Ethylene-octene copolymerized elastomer is a synthetic thermoplastic elastomer [1,2] with a significant narrow molecular weight distribution and six-carbon short-chain branches [3,4,5]. Ethylene in the copolymer can be crystallized, and this part of crystallization serves as a physical crosslinking point to provide material strength [6,7,8]. The amorphous portion of the random ethylene-octene segment contributes to the elasticity [6,9]. Its flexibility and mechanical properties, like synthetic rubber, combined with the melt processability, make the ethylene-octene elastomer a very good material with many industrial applications [10,11,12,13,14]. However, its large permanent deformation and other disadvantages limit its application. In certain applications, EPDM is commonly used to improve the elasticity or toughness of plastic materials [15,16]. EPDM has been blended with POE to improve the properties of POE. For EPDM/POE blending, substantial studies have been carried out on the mechanical properties and the crosslinking methods [15,16,17]. Modified POE with crosslinked EPDM using the dynamic crosslinking method [17] can obtain good mechanical properties. The processing procedures are the same as the ones used to prepare PP/EPDM thermoplastic vulcanizates (TPV). 

However, the POE/EPDM blend is different from typical TPV. For polypropylene (PP)/EPDM TPV, EPDM was crosslinked and dispersed as small particles in the PP matrix and high strength is derived from the PP crystals [18]. Random ethylene-octene copolymer differs from PP in two aspects. One aspect is that POE, like EPDM, can also be crosslinked, whereas PP cannot [19]. Another aspect is that POE/EPDM is more compatible than PP/EPDM blends [17,20]. Therefore, a POE/EPDM blend using the dynamic crosslinking method is not a typical TPV. Wang et al. [17] used RPA and a phase-contrast microscope to investigate the effect of dynamic crosslinking by blending POE thermoplastic elastomers with EPDM. The mechanical properties of POE/EPDM blends were improved, and the addition of EPDM effectively improved the permanent deformation of POE [17]. However, there are still some fundamental issues related to dynamic crosslinked materials with components that are probably all crosslinked, which are important but lack of study, such as how dynamic crosslinking influences the molecular structures, the compatibility of the two materials before and after crosslinking, and its effects on crystallization dynamics and mechanical properties. Studying these elements is important for industrial applications.

In the present work, partially crystalline EPDM and an ethylene-octene random copolymer elastomer (ENGAGE™ 8480 Polyolefin Elastomer) are used to investigate these effects on POE/EPDM blends. The non-isothermal crystallization dynamics and thermal properties of POE/EPDM blends before and after dynamic crosslinking are investigated with differential scanning calorimetry (DSC). The molecular structure of the blends and the influence of microstructural changes from crosslinking are studied with small-angle X-ray scattering (SAXS). The dynamic mechanical property and the structure-property relationship are also discussed.

## 2. Experimental Part

### 2.1. Material and Sample Preparation

The polyolefin elastomer (POE copolymer) selected for this study was ENGAGE™ 8480 polyolefin elastomer, which is an ethylene-octene copolymer with 20 wt% octene content supplied by Dow Chemical Company (Midland, TX, USA). It has an M_w_ of 94500 g/mol, M_w_/M_n_ ~ 2.4, a melt flow rate (MFR) of 1.0 g/10 min (ASTMD-1238, 230 °C, 2.160 kg), a density of 0.902 g/cm^3^, a viscosity ML_1+4_ (at 121 °C) of 18, and a melting point of 98 °C. The random ethylene-octene copolymers investigated in this study do not form the typical structure of lamellar stacks, but rather mixed individual crystals with folded chains and fringed micellar crystals. DCP was selected to crosslink POE with 2 wt% dose of EPDM. 

EPDM was purchased from SABIC Co., Ltd. (Riyadh, Saudi Arabia), which has an ethylene content of 69%, a third monomer content of 2.8% (ENB), and a Mooney viscosity of 67 Mu (ASTMD-1646, ML_1+4_, 125 °C).

Dicumyl peroxide (DCP) (Perkadox-BC-40B-PD) with an active peroxide content of 40%, a temperature at which half-life time (t_1/2_) is 1 h of 138 °C, and a specific gravity of 1.53 g/cm^3^ at 23 °C was obtained from AkzoNobel chemical company (Ningbo, China).

The POE/EPDM blend: A two-step method was used to prepare the POE/EPDM blend, which reduced POE crosslinking. First, EPDM matrix and DCP were mixed uniformly on a two-rolling mill at 25 °C. After 3 min of mixing time, the pre-blends were removed from the mixer. In the second step, POE matrix and the pre-blends were mixed uniformly in a torque rheometer at 110 °C for about six minutes at 110 °C and a rotor speed of 40 rpm to ensure the homogeneous distribution of the blends. Then, the temperature was raised to 165 °C, and the compound was cured during compounding. Crosslinked EPDM was prepared using the same method. The formulas of POE/EPDM blends are listed in Table 1. 

### 2.2. Thermal Analysis

Thermal behaviors of all the samples were studied via DSC (Q100 system, TA, New Castle, DE, USA) equipped with a liquid nitrogen cooling accessory. The tested sample was sealed in an aluminum holder and was heated up from 0 °C to 150 °C at a rate of 10 °C/min with nitrogen gas, which is used as an environment in a system and for displacing oxygen. The samples were then cooled to 0 °C at 10 °C/min to investigate their non-isothermal crystallization. The samples were subsequently heated back to 150 °C at the same rate to investigate their melting behaviors.

### 2.3. Dynamic Mechanical Analysis (DMA)

Dynamic mechanical analysis was carried out in a Diamond DMA machine (Waltham, MA, USA). The dynamic behavior of POE is strongly influenced by crystalline state, which is affected by thermal history [21,22]. Therefore, the system was cooled to −70 °C from room temperature to avoid the effect of thermal history effects. Specimens were tested in the dynamic tensile mode at a frequency of 1 Hz from −80 °C to 80 °C with a heating rate of 3 °C min^−1^. Sample dimensions were 10 × 50 × 1 mm.

### 2.4. In Situ Tensile and Synchrotron SAXS Measurements

A Linkam tensile device (Tadworth, UK) was used for the in situ SAXS studies. The initial sample length L_0_ (the length between the two clamps) was 16 mm, with sample width and thickness of 4 mm and 1 mm, respectively. The stretching rate for the in situ experiment was 10 mm/min, with a maximum elongation of 375% due to the travel limitation of the tensile device used. 

SAXS measurements were performed at the synchrotron beamline 1W2A of the Beijing Synchrotron Radiation Facility, Beijing, China. The energy of the X-ray radiation was 8.052 keV, resulting in a wavelength of 0.1542 nm. The sample (0.1 × 1 × 3 cm^3^ dimensions) was mounted onto a portable tensile tester at the beam line. For SAXS measurement, the beam-stop size chosen was 4 mm, and the distance between the sample and the detector was 1591.02 mm. At this distance, the effective scattering vector q was defined as q = 4πsinθ/λ, where λ is the X-ray wavelength and θ is half of the scattering angle (2θ). The SAXS patterns were recorded with a two-dimensional Mar CCD detector with an active area of 165 mm diameter. The collected data were corrected for air background before any analysis. Data processing was performed with the computer program “XPolar.” The SAXS patterns were normalized to the primary beam intensity and corrected for background scattering.

### 2.5. Mechanical Testing

The sample was prepared with a thickness of 1 mm using a cutting machine for dumbbell-shaped sample bars with dimensions of 75 mm length, 25 mm neck length, and 4 mm neck width. The deformation of the sample was performed with an Instron 3365 tensile apparatus. The original length L_0_ between the Instron jaws was 20 mm. The stretching was carried out in a symmetrical mode, where the detection spot on the sample remained fixed in space. The experiments were carried out at room temperature (25 °C). A constant extension rate (100 mm/min) was applied to the specimen throughout the deformation process. 

## 3. Results and Discussion

### 3.1. The Variation of Torque for POE/EPDM Blend Crosslinking Promoted by DCP

**Figure 1:**
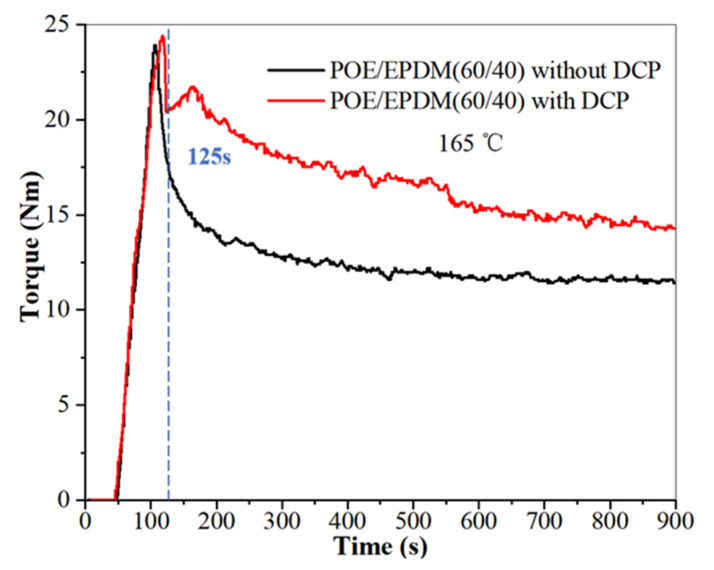
Variations of torque of POE/EPDM blends with and without DCP at 165 °C in a torque rheometer.

Dynamic crosslinking is shear stress acting in the rubber phase during rubber crosslinking, which leads to the rubber phase becoming small particles and dispersing in the plastic phase (take PP as an example). In this paper, this method was used to prepare a crosslinked POE/EPDM blend. However, POE is different than PP, which can also be crosslinked by DCP [23]. Therefore, to reduce POE crosslinking and make DCP in the EPDM phase as much as possible, a two-step method was used to prepare crosslinked POE/EPDM blend. DCP was mixed with EPDM at room temperature in the first step. A POE/EPDM blend without adding EPDM was also prepared for comparison. The variations in the torque and temperature as a function of mixing time during dynamic blending in the torque rheometer are shown in Figure 1.

Figure 1 shows that the variation in torque with shearing time in the torque rheometer for the blends with and without DCP was different. At the initial stage, the torque decreased with an increase in mixing time for both of the blends because of the melting of the blends. The torque of the blend with DCP reached a local minimum at 125 s and then rose dramatically until it reached a peak at 180 s, indicating a rapid crosslinking with time. The torque of the blend without DCP decreased with shearing time due to the shearing effect. The higher torque of the blend with DCP than the blend without DCP resulted from the crosslinking of EPDM and probably part of POE.

### 3.2. Non-Isothermal Crystallization of the Neat POE and POE/EPDM Blends with DSC

**Figure 2:**
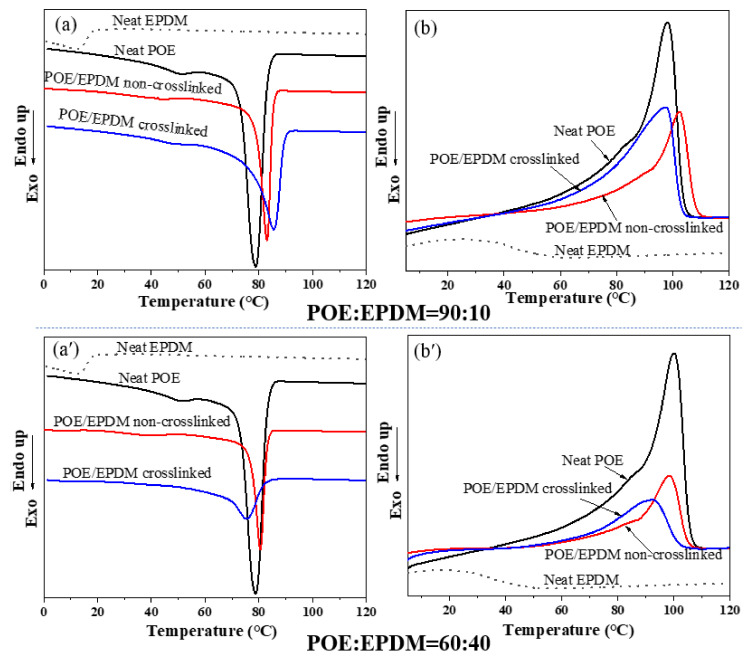
DSC cooling (10 °C/min) thermograms and the subsequent melting (10 °C/min) thermograms of neat POE, neat EPDM, dynamic crosslinked POE/EPDM, and non-crosslinked POE/EPDM: (**a**,**a′**) cooling thermograms; (**b**,**b′**) the subsequent melting thermograms. Crosslinked blends show lower melting temperature, indicating that the crystals are smaller.

The crystal structure of random ethylene-octene copolymers (engage 8480 used in this study) have small fringed micellar crystals, which play an important role in the properties. Due to the crystallization of POE, molecular chain slip is common, resulting in significant permanent deformation of the materials [24]. The addition of EPDM can affect the crystallization of POE, which can then affect other mechanical properties. DSC was used to investigate the non-isothermal crystallization and melting behavior of the neat POE, neat EPDM, and POE/EPDM blends with and without dynamic crosslinking. The cooling and second heating trace are shown in Figure 2a–b′, respectively.

EPDM (ethylene content of 69%) with an ethylene content higher than 64–65 wt% is a semi-crystalline material at room temperature of polyethylene (PE) at high ethylene concentrations [25]. The neat EPDM showed a weaker crystallization peak at 11 °C and a weaker melting peak at 15 °C than neat POE (Figure 2a), which can be attributed to ethylene segment crystallization, indicating that the EPDM had a weaker crystallization ability. Figure 2a shows that neat POE demonstrated two crystallization peaks at 50 °C and 79 °C, which were the crystallization of relatively short ethylene chain segments and long ethylene chain segments in POE, respectively. The crystallization peak of EPDM in blends became indistinct after blending POE and EPDM in the torque rheometer (without crosslinking) (Figure 2a,a′). The weak crystallization peak of the blends with 10 wt% EPDM and 40 wt% EPDM moved to a lower end than neat POE (neat POE at 50 °C) at 43 °C and 40 °C, respectively, and the main sharp crystallization peak moved to a higher temperature than that of neat POE. This result can be interpreted as follows. EPDM offers an amorphous phase for the blends, which improves the mobility of the molecular chains, leading to the chain segments with higher crystallization adjusting more easily and crystallizing at a higher temperature, whereas the chain segment with worse crystallization ability and with a high octane content is easy to exclude from the amorphous phase, leading to phase separation and crystallize separately at a lower temperature. The 2 °C lower crystallization peak of the blend with 40 wt% EPDM than the blend with 10 wt% EPDM suggests that too much EPDM hinders the mobility of molecular chains, leading to the crystallization ability of POE being suppressed by EPDM.

After dynamic crosslinking, the crystallization behavior of EPDM/POE blends significantly changed. The chain segments of the -CH_2_- structure contributed significantly to the crosslinking [26]. After crosslinking, the crystallization peak from EPDM disappeared (Figure 2a,a′), indicating that crosslinking destroyed the short-range order of PE in the EPDM molecular chains. The initial crystallization temperature of the blends with 10 wt% EPDM crosslinked (85 °C) was higher than non-crosslinked blends (83 °C). The slightly higher initial crystallization temperature of the dynamic crosslinked blend than the non-crosslinked sample was presumably due to easier nucleation within a crosslinking network [23], which acts as a crystal nucleus and thus induces crystallization. Compared with the non-crosslinked blends, the crystallization peaks of dynamic crosslinked blends were wider. This result can be attributed to the effect of the crosslinking network, which limits the chain mobility in the crystal region.

The crystallization peak position of the main peak differed significantly between dynamic crosslinked blends containing 10 wt% and 40 wt% EPDM. The crystallization peak temperature (87 °C) of the blend containing 10 wt% EPDM was higher than that of the neat POE, whereas the crystallization temperature for the blends containing 40 wt% EPDM was lower than for neat POE. Meanwhile, when 40 wt% EPDM was added, the crystallization of short ethylene chain segments and some PE molecules with high octane content in POE disappeared, as shown in Figure 2a′. These results also indicate that too much crosslinked EPDM suppresses the crystallization ability of POE.

Figure 2b,b′ show the secondary heating traces of neat POE, neat EPDM, and blends with and without crosslinking. The crystallinity, evaluated based on the theoretical heat of fusion ΔH0 m for 100% crystallized polyethylene being 293 J/g [23], is also listed in Table 2 for all the samples. As listed in Table 2, the dynamic crosslinked blend with 10 wt% EPDM showed a higher crystallinity than non-crosslinked blends, suggesting that although the crosslinking limits the mobility of the molecules, the crosslinking network acts as nucleation and thereby improves the crystallization ability, leading to higher crystallinity. However, the crosslinked blend with 40 wt% EPDM showed lower crystallinity than non-crosslinked blends, which is opposite to the result of 10 wt% EPDM blends, indicating that the effect of decreased chain mobility from EPDM crosslinking network is dominant and suppresses the crystallization ability.

Moreover, the non-crosslinked blends showed a main sharp melting peak at 100 °C and a weak melting peak at 84 °C (Figure 2b,b′), which can be attributed to the main fraction of long ethylene segments with low octane content and relatively short ethylene segments with high octane content, which is accordance with the neat POE. However, the weak melting peak disappeared for the crosslinked blends, indicating that the crosslinking network significantly affects the crystallization ability, and the suppression of the network on chain mobility weakens the effect of the octane content of POE on crystallization ability.

The above study demonstrates that the high mobility of EPDM in POE/ EPDM blends improves the crystallization ability to some extent, evidenced by the higher crystallization temperature than for neat POE. For the blends with 10 wt% EPDM after being crosslinked, the nucleation and crystallization started at a higher temperature, which was presumably due to easier nucleation from the crosslinking network. However, too much EPDM hindered the mobility of POE and thus exhibited a lower initial crystallization temperature. The crosslink network limited the chain mobility, as evidenced by the wider crystallization peak of crosslinked blends and lower crystallinity temperature of EPDM crosslinked POE than non-crosslinked. Higher crystallinity was observed for the dynamic crosslinked 10 wt% EPDM blends than for neat POE, but lower for the dynamic crosslinked 40 wt% EPDM blends, suggesting that a few crosslinking networks can induce the crystallization, whereas too many crosslinking networks (40 wt% EPDM in this study) limit the mobility of the molecules and thereby suppress the crystallization process, leading to lower crystallinity.

### 3.3. Molecular Structure of POE/EPDM Blends with and without Dynamic Crosslinking

For the random ethylene-octene copolymers investigated in this study, mixed individual crystals with folded chains and fringed micellar crystals were formed. In this study, synchrotron SAXS was used to investigate the structure of neat POE and blends with and without dynamic crosslinking at the nanoscale.

**Figure 3:**
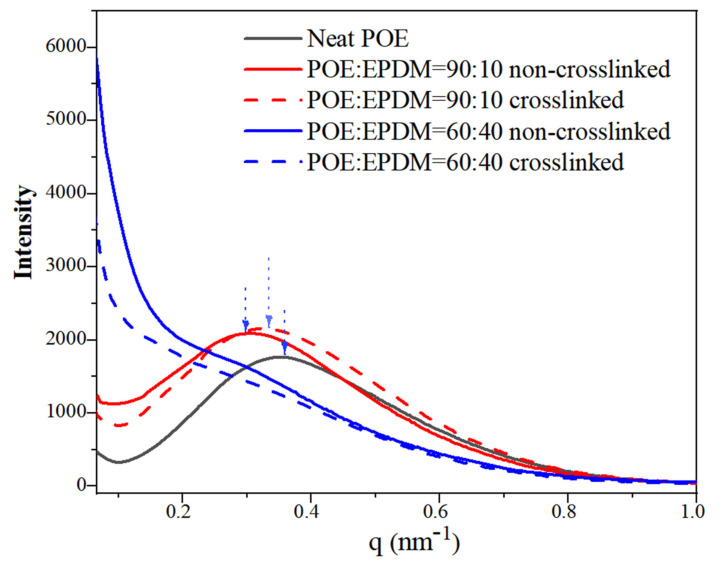
Linear SAXS profiles of neat POE and POE/EPDM blends: neat POE (black line), POE:EPDM = 60:40 non-crosslinked (blue line), POE:EPDM = 60:40 crosslinked (blue dotted line), POE:EPDM = 90:10 non-crosslinked (red line), POE:EPDM = 90:10 crosslinked (red dotted line).

As shown in Figure 3, neat POE (black line) exhibited a main well-defined scattering peak around q at 0.37 nm^−1^, corresponding to the fringed micellar crystals of neat POE. For the blend with 10 wt% EPDM, the characteristic scattering peaks still exhibited good shape, but shifted to a lower peak position, indicating that the addition of a small amount of EPDM enlarged the inter-distance of POE micellar crystals. Therefore, it can be inferred that a small amount of EPDM can enter the phase region of POE, and hence enlarge the spacing of POE micellar crystals. On the other hand, after dynamic crosslinking, the scattering peak of the blend shifted back to a higher peak position, indicating a smaller average inter-distance. After crosslinking, it can be seen from the melting traces in Figure 2b that the melting point of the blends decreased, indicating that smaller crystals were formed. Therefore, the inter-distance of neighboring crystals becoming smaller after crosslinking is presumably caused by the crystal fragmentation after crosslinking.

The change in peak position was attributed to the change in the average inter-distance of neighboring crystals (or long period) in the sample, which can be quantitatively evaluated from the Lorentz-corrected SAXS profiles of the sample [27]. The scattering peak of the blend with 10 wt% non-crosslinked EPDM shifted to a lower q end than neat POE, suggesting that the average inter-distance of neighboring crystals in stretching direction becomes larger.

After blending 40 wt% EPDM, the scattering signal at a low q angle (0–0.3 nm^−1^) was very high. In this blending system, the density of the POE amorphous phase and EPDM was 0.80 g/ cm^3^ and 0.89 g/cm^3^, respectively, leading to a density contrast between EPDM and POE. Since the scattering intensity is proportional to the square of the density contrast, the strong scattering intensity was probably caused by the POE-EPDM phase separation. Meanwhile, the main crystallization scattering peak of POE almost disappeared and the shape became a shoulder, indicating too much EPDM decreased the crystallization regularity of POE. After crosslinking, the scattering signal at a low q angle decreased obviously, indicating that the POE/EPDM phase separation decreased. Moreover, the scattering peak of POE was still not well defined, suggesting that the regularity of crystallization was low.

**Figure 4:**
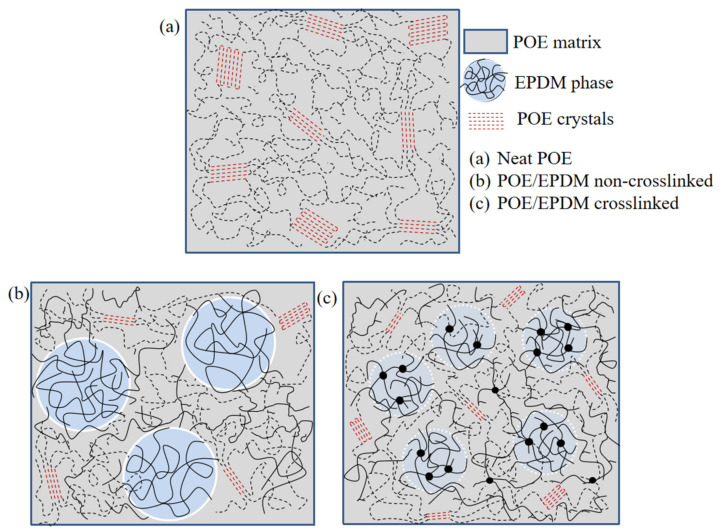
Structural depiction of POE and the blends. (**a**) Neat POE. It forms mixed individual crystals with folded chains and fringed micellar crystals (red dotted line). (**b**) POE/EPDM blends. Although EPDM has a certain compatibility with POE, the blend system still forms a phase-separation structure. (**c**) Dynamic crosslinked POE/EPDM blends (take POE:EPDM = 60:40 as the example).

On the basis of these results, a structural depiction of the microstructures for pure POE and blends with and without crosslinking is shown in Figure 4. It can be seen in Figure 4a that neat POE was a mix of individual crystals with folded chains and fringed micellar crystals. Although EPDM had a certain compatibility with POE, the POE-EPDM blend system still formed a phase separation structure, and the large phase regions are shown in Figure 4b. This result can be interpreted as follows. During the blending process in the melting state, POE and EPDM molecular chains entangle, but in the later cooling process, the ethylene segments in POE crystallize and EPDM molecules “clump” with each other at the same time, thus forming a two-phase structure, which is consistent with the higher scattering signal than for neat POE at a small q angle in the SAXS result (Figure 3). However, in this case, EPDM had a limited impact on the crystallization of the ethylene chain segments in POE, which is consistent with the similar sharp crystallization peak of the blends with neat POE in the DSC cooling curves (Figure 2). It can be seen in Figure 4c that the two-phase structure was weakened after dynamic crosslinking, which can be illustrated as follows. Dynamic crosslinking mainly occurred in EPDM molecules as a result of the two-step processing method. Nonetheless, a small number of DCP molecules were still distributed in POE molecules and led to POE crosslinking together with EPDM. On the other hand, under the dynamic shearing and cooling process, this part of POE molecular chains was drawn into the EPDM phase region by a large number of “bonded” EPDM molecular chains, resulting in a decrease in the density contrast between the two phases. Therefore, the scattering signal of SAXS at a low q angle decreased obviously after crosslinking (see Figure 3). Besides, more EPDM molecules being in the POE phase region can be attributed to the effect of crosslinking points. The EPDM molecules and crosslinking points in the POE phase region hindered the crystallization of POE, and the crosslinking points hindered the movement of POE molecular chains, resulting in the crystal becoming smaller, which is consistent with the lower melting point of the crosslinked blends in the DSC curve (Figure 2). The crystal fragmentation led to smaller average inter-space between crystals than that of non-crosslinked blends, which is in accordance with the smaller long-period of the dynamic crosslinked blend than the non-crosslinked blend obtained in Figure 3.

### 3.4. In Situ Tensile and Synchrotron SAXS Study on Packing Structure of Crosslinked POE/EPDM Blends during a Uniaxial Stretching Process

**Figure 5:**
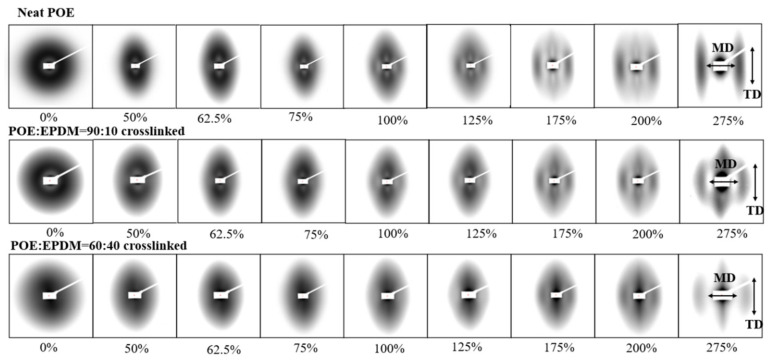
The 2D SAXS patterns of the dynamic crosslinked blends (POE:EPDM = 90:10 and POE:EPDM = 60:40) and neat POE.

Figure 5 shows the 2D SAXS pattern of neat POE and the POE/EPDM blend with 10 wt% EPDM (“blend”) collected in the MD direction at different strains. It can be seen from Figure 5 that the SAXS patterns of neat POE and the blends before deformation showed a broad isotropic ring, indicating that there was no preferred crystal orientation for the non-deformed samples. For all the samples, the SAXS pattern changed dramatically with strain and an additional scattering pattern, such as “11,” was present along the MD direction, in addition to the scattering pattern “8” aligned in the TD direction at about 100–s125% strain, indicating the formation of new crystals [23].

**Figure 6:**
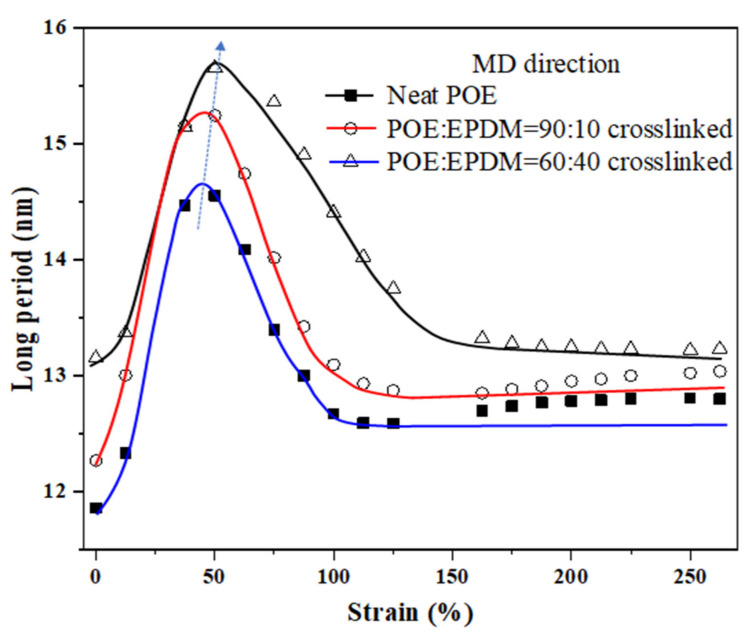
The SAXS profiles in the MD direction for the neat POE, crosslinked POE:EPDM = 90:10, and crosslinked POE:EPDM = 60:40 at different strains. In the stretching direction, the peak position of the long period is located at higher strains for the crosslinked blends.

The “long period” is used to represent the change in the averaged inter-distance of neighboring crystals in the sample, which can be quantitatively evaluated from the Lorentz-corrected SAXS profiles of the sample [27]. It can be seen from Figure 6 that in the stretching direction, the long period first increased with strain and then decreased, indicating that when the strain continued to increase, large crystals were fragmentated. Besides, the new crystals were formed at about 100-125% strain, as shown in Figure 6, which is in accordance with the “11” scattering pattern in Figure 5. Finally, the long period in Figure 6 was stable. Compared with neat POE, with an increase in strain, the maximum value of the long periods for the blends with 10 wt% EPDM and 40 wt% EPDM appeared at higher strains (53% and 69% strain, respectively) than for neat POE, indicating that crosslinked EPDM can hinder the damage of crystals, leading to the crystals being fragmentated later. The above result indicates that crosslinked EPDM hindered the destruction of crystals and led to the delayed destruction of crystals.

### 3.5. Dynamic Mechanical Behavior

**Figure 7:**
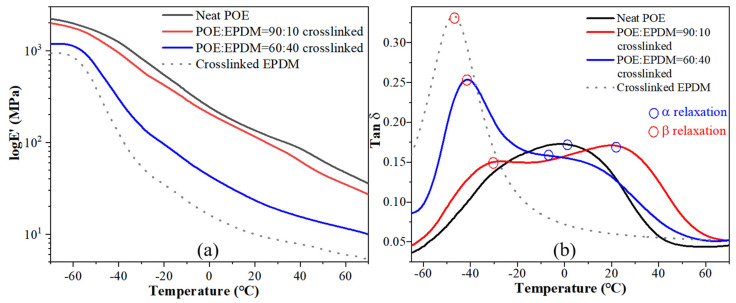
Storage modulus (**a**) and tan δ (**b**) of neat POE and dynamic crosslinked POE/EPDM blends.

The dynamic mechanical properties of dynamic crosslinked POE/EPDM blends are shown in the form of the storage modulus and loss tangent (tan δ) in Figure 7. POE exhibited two relaxations, conventionally identified as α and β relaxation in decreasing temperature order at temperatures higher than −100 °C, which has been extensively studied [28,29,30,31]. The α relaxation is usually identified with the crystalline phase of polyethylene and is attributed to the translation of chain segments along the crystalline axis [21,22]. β relaxation is assumed to be related to the movement of the amorphous region, namely, glass transition for elastomers. Figure 7a shows that the storage modulus of blends gradually decreased with an increase in EPDM fraction, attributed to the soft characteristic of EPDM [4].

Figure 7b shows the relationship between the loss factor (tan δ) and temperature of neat POE and crosslinked POE/EPDM blends. It is shown in Figure 7b that neat POE showed a broad peak, which is different from the narrow peak of EPDM. The β relaxation transition peak of neat POE was not obvious (about −25 °C) and was covered by the α peak related to crystallization. Crosslinked EPDM had a narrow β relaxation transition peak (glass transition peak). The blend with 10 wt% EPDM showed two distinct conversion peaks, corresponding to β conversion of EPDM (including POE) and α conversion of POE. The single β conversion position of the blend with 10 wt% EPDM shifted to a lower temperature direction (−30 °C) compared to neat POE, but higher than the β conversion temperature of crosslinked EPDM, indicating that EPDM and POE had good compatibility. The peak position of α conversion for the blends with 10 wt% EPDM moved to higher temperatures (21 °C) than for neat POE (about 5 °C), indicating the molecular chains entered or existed at the edge of the crystallization region of POE. Although the crystallinity of the blends decreased, more fine microcrystalline regions were formed, which increased the α transformation temperature. For the blend with 40 wt% EPDM, the shape of the α conversion peak became a shoulder, indicating too much EPDM decreased the crystallization regularity of POE, resulting in a decrease in the α conversion peak and α conversion temperature.

The above study shows that POE and EPDM have good compatibility in POE/EPDM blend using the dynamic crosslinking method, as evidenced by the β conversion tempe-ature lower than for POE and higher than for EPDM for blends containing 10 wt% and 40 wt% EPDM, as well as a higher α conversion temperature than for neat POE.

### 3.6. Tensile Properties of the POE/EPDM Composites during a Uniaxial Stretching Process

**Figure 8:**
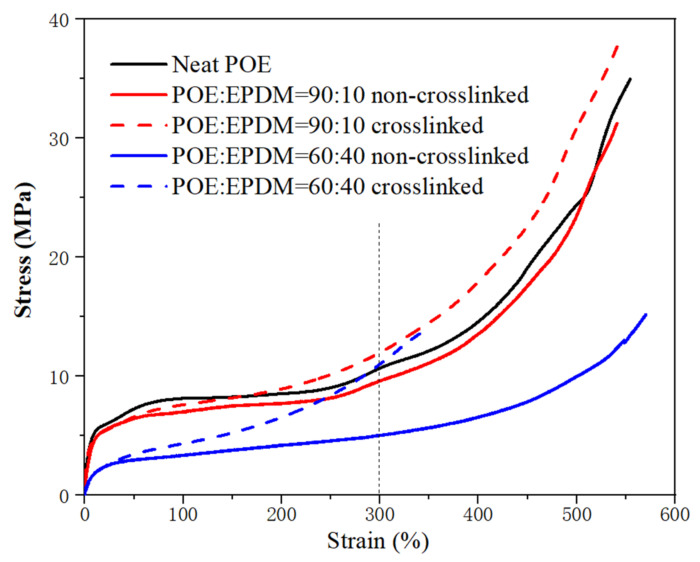
The stress-strain curves (up to break) of neat POE and POE/EPDM blends.

The stress-strain curves of the POE/EPDM blends during uniaxial deformation are shown in Figure 8. It can be seen from Figure 8 and Table 3 that the elongation at break of non-crosslinked blends did not decrease compared to neat POE, despite the phase separation structure of POE and EPDM existing, as discussed in above SAXS result, indicating that the phase interfaces between POE and EPDM were good (Figure 3). The tensile strength of the 10 wt% and 40 wt% EPDM blends was 31.3 MPa and 15.2 MPa, respectively, and both were lower than neat POE (34.9 MPa), which was a result of the lower strength of the EPDM rubber.

It was noticed that after dynamic crosslinking, the stress at 300% strain of the blends was significantly higher than for non-crosslinked blends, as shown in Figure 8, which was attributed to the better strength of the crosslinking network and the later destruction of crystals. However, the elongation at break decreased for the dynamic crosslinking blend with 40 wt% EPDM compared to the non-crosslinked sample. This result can be interpreted as follows. As known from above, the POE/EPDM phase separation decreased after dynamic crosslinking. Therefore, the decrease of elongation can be attributed to the effect of POE and EPDM crosslinking in the blend system. On the other hand, for the dynamic crosslinked blend with only 10 wt% EPDM, the elongation was almost the same as the non-crosslinked sample, indicating the influence of the better compatibility of EPDM and POE having a dominant effect compared to the effect of crosslinking. The permanent set of crosslinked blends was improved compared to the non-crosslinked samples. It can also be seen in Table 3 and Figure 8 that the blend with 10 wt% EPDM showed better tensile properties. This result further proves that the addition of a small amount of EPDM and dynamic crosslinking can improve the mechanical properties of POE.

## 4. Conclusions

In the present work, the effects of dynamic crosslinking on the non-isothermal crystallization, compatibility, and mechanical properties of POE/EPDM blends were investigated. To reduce the crosslinking of neat POE, EPDM and DCP were mixed first during compounding. The good mobility of EPDM improved the crystallization ability of POE before crosslinking, as evidenced by the higher crystallization peaks of the blends than of neat POE. For the blends with 10 wt% EPDM after being crosslinked, the nucleation and crystallization started at a higher temperature, which was presumably due to easier nucleation from the crosslinking network. However, too much EPDM hindered the mobility of POE and thus had a lower initial crystallization temperature.

The non-crosslinked blends had a two-phase separation structure, but it was weakened after dynamic crosslinking, indicating better compatibility of POE and EPDM than non-crosslinked, as evidenced by the lower scattering signal at a low q angle than for the non-crosslinked blend from the SAXS study. The good compatibility of POE and EPDM of the dynamic crosslinked POE/EPDM blend was evidenced by a β conversion temperature lower than POE and higher than EPDM for blends containing 10 wt% EPDM, as well as a higher α conversion temperature than for neat POE, as obtained from DMA.

The elongation at break of the non-crosslinked blends did not decrease compared to pure POE, despite the phase separation structure of the blend existing, indicating that the phase interfaces between POE and EPDM were good. However, the elongation at break decreased for the dynamic crosslinking blend with 40 wt% EPDM compared to the non-crosslinked sample, which can be attribute to the effect of POE and EPDM crosslinking in the blend system. The stress at 300% strain of the dynamic blends was significantly higher than for the non-crosslinked blends, which can be attributed to the better strength of crosslinking network. On the other hand, for the dynamic crosslinked blend with only 10 wt% EPDM, the elongation was almost the same as for the non-crosslinked sample, indicating that the influence of the better compatibility of EPDM and POE had a dominant effect compared to the effect of crosslinking.

## Figures and Tables

**Table 1:** The formulas of neat POE, crosslinked EPDM, and POE /EPDM blends.

Sample Formula	Engage 8480	EPDM	DCP
Neat POE	100	0	0
POE:EPDM = 90:10 non-crosslinked	90	10	0
POE:EPDM = 90:10 crosslinked	90	10	0.2
POE:EPDM = 60:40 non-crosslinked	60	40	0
POE:EPDM = 60:40 crosslinked	60	40	0.8
Crosslinked EPDM	0	100	2.0

**Table 2:** Crystallization temperature, enthalpy of fusion, crystallinity, and melting point of neat POE and POE/EPDM blends with and without dynamic crosslinking.

Sample	Crystalline Temperature (°C)	△H_f_ (J/g)	Crystallinity (wt%)	Melting Point (°C)
Neat POE	79	125.8	42.9	100
POE:EPDM = 90:10 non-crosslinked	83	77.4	29.4	99
POE:EPDM = 90:10 crosslinked	86	94.0	35.7	99
POE:EPDM = 60:40 non-crosslinked	81	65.2	37.1	99
POE:EPDM = 60:40 crosslinked	75	58.1	33.0	92

**Table 3:** Mechanical properties of neat POE and the POE/EPDM blends.

Sample	Tensile Strength/MPa	Elongation at Break/%	Tensile Stress at 100%/MPa	Tensile Stress at 300%/MPa	Permanent Set of Elongation Break/%
Neat POE	34.9	554	8.1	10.7	420
POE:EPDM = 90:10 non-crosslinked	31.3	541	7.0	9.6	400
POE:EPDM = 90:10 crosslinked	37.8	541	7.6	12.0	370
POE:EPDM = 60:40 non-crosslinked	15.2	570	3.3	5.0	285
POE:EPDM = 60:40 crosslinked	13.8	345	4.3	11.0	168

## Data Availability

All the data are available within this manuscript.
